# Supplementary material for: Preparation and Properties of Corn Starch/Chitin Composite Films Cross-Linked by Maleic Anhydride
Source: Polymers (Basel). 2020 Jul 19;12(7):1606. doi: 10.3390/polym12071606 (PMC7408147; doi:10.3390/polym12071606)
Supplement: Supplementary file 1 [file polymers-12-01606-s001.pdf]

Supplmentary Materials

Preparation and properties of corn starch/chitin composite films cross-linked by maleic anhydride

(a)

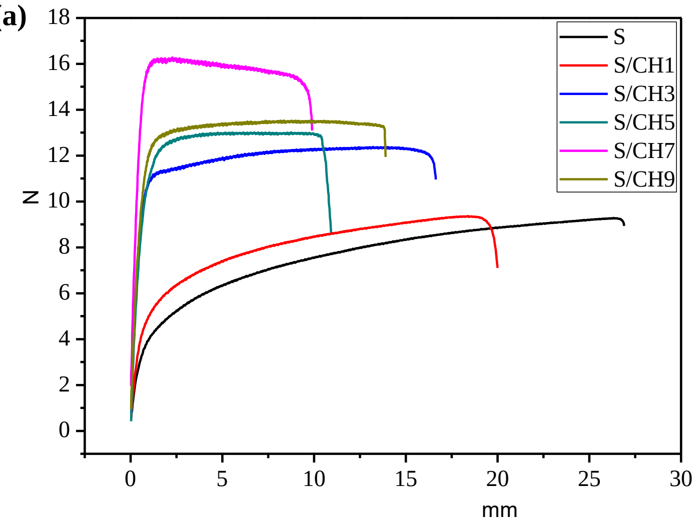

(b)

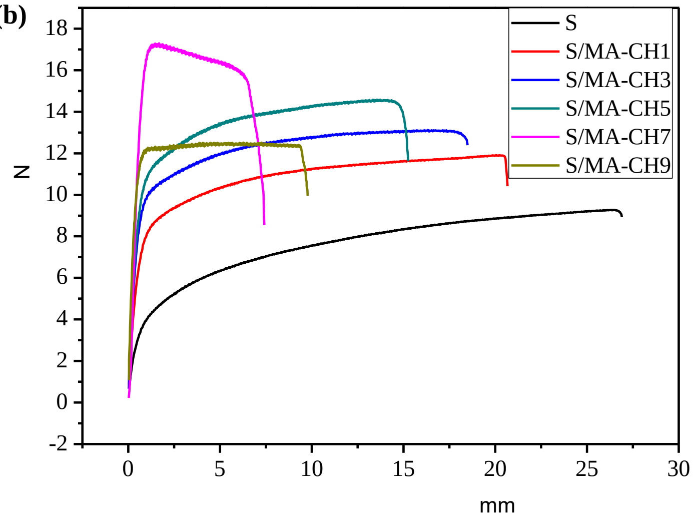

Figure S1. Tensile test curve of (**a**) S films, S/CH 1% films, S/CH 3% films, S/CH 5% films, S/CH 7% films, S/CH 9% and (**b**) S films, S/MA-CH 1% films, S/MA-CH 3% films, S/MA-CH 5% films, S/MA-CH 7% films and S/MA-CH 9% films.
